# Social prescribing in primary care for people living with dementia: a qualitative exploration of different roles and services in England

**DOI:** 10.1186/s12875-025-03011-9

**Published:** 2025-11-07

**Authors:** Aimee Pick, Emma Wolverson, Jane Cross, Chris Fox, Esme Moniz-Cook, Joanne Reeve, Kritika Samsi, Louise Robinson, SPLENDID Collaboration, Louise Allan, Anthony Avery, Katherine Bradbury, Anne Irvine, Jessica Marshall, Antonieta Medina-Lara, Nia Morrish, Martin Orrell, Evie Papavasiliou, Fiona Poland, Marie Polley, Amy Rathbone, George Rook, Euan Sadler, Lee Shepstone, Sarah Walker

**Affiliations:** 1 Newcastle University, Newcastle Upon Tyne, NE1 7RU UK; 2 Geller Institute of Ageing and Memory, University of West London, London, UK; 3 Dementia UK, London, UK; 4 University of East Anglia, Norwich, Norfolk, NR4 7TJ UK; 5 University of Exeter, Stocker Road, Exeter, EX4 4PY UK; 6 University of Hull, Cottingham Road, Hull, HU6 7RX UK; 7 Kings College London, London, UK

**Keywords:** Social prescribing, Dementia, Link worker, Primary care

## Abstract

**Background:**

Dementia is a global public health challenge with the number of people living with the condition rapidly rising. Social prescribing in primary care has emerged as a person-centred approach connecting individuals with community support. It is increasingly explored for its potential to support people with complex needs, yet its role in dementia care remains uncertain. This study aimed to explore current provision of social prescribing for people living with dementia across England, identifying relevant aspects for dementia care, with particular focus on generic and specialist services.

**Methods:**

Semi-structured interviews were conducted with regional leads of social prescribing services and social prescribing link workers (SPLWs) across England. Data were analysed using template analysis to identify key themes.

**Results:**

Twenty-two participants were interviewed: ten social prescribing regional leads, four generic SPLWs commissioned to work with people aged > 18 years, and eight SPLWs working exclusively or partly with a specific adult population.

Four themes were identified: family carer engagement key to supporting people living with dementia; service rather than person-centred care; the dominance of dementia in influencing support; and strategies for success: dementia centred social prescribing.

Participants identified the central role of family carers in facilitating access to social prescribing, highlighting that carer support was often essential. Generic social prescribing frequently followed a ‘service-led’ approach, with service constraints negatively influencing interactions. Dementia was often perceived as the dominant support need, potentially marginalising individuals within broader social prescribing services. Despite these challenges, participants with more role flexibility, and/or more experience of dementia, demonstrated a range of successful strategies, illustrating the potential of social prescribing for people living with dementia.

**Conclusion:**

SPLWs perceive that social prescribing has potential to play a key role in support for people living with dementia and family carers. While its core principles align well with dementia care, our findings suggest a social prescribing model more tailored to the needs of people living with dementia, or additional dementia-specific training for generic SPLWs, may prove more effective. Further research is needed to assess the impact of these approaches, especially for people living with dementia without access to family carers.

**Supplementary Information:**

The online version contains supplementary material available at 10.1186/s12875-025-03011-9.

## Introduction

Dementia is a global public health issue as ageing societies lead to increasing prevalence of the condition worldwide [1]. In England, there were 944,000 people living with dementia in 2022; predicted to be over 1.5 million by 2040 [2]. The complexities associated with dementia include the effects of cognitive impairment, the impact of age-related health co-morbidities, social exclusion as well as economic demands on people living with dementia, their families and health and social care systems [3–8]. The social health paradigm as applied to dementia care is a growing area of research and knowledge [9, 10]. Within this paradigm, efforts to counteract stigma and ‘othering’ among those recently diagnosed by facilitating social opportunities may yield positive outcomes in preventing some of the well-known aspects of social isolation in people living with dementia [8, 11, 12]. Social prescribing is a non-pharmacological approach defined as "a means for trusted individuals in clinical and community settings to identify a person who has non-medical, health-related social needs and to subsequently connect them to non-clinical supports and services within the community by co-producing a social prescription,” (p8) [13]. Social prescribing aims to improve health and wellbeing outcomes and reduce burden on health services [14, 15]. In England, it is a key component of the National Health Service (NHS) augmented by a financial commitment that by 2023, every primary care General Practice (GP) had access to a social prescribing link worker (SPLW) [16]. In dementia, social prescribing’s focus on person-centred care aligns with primary care goals and policy initiatives to promote functional capabilities and independence for people living with dementia in the community [17]. The emphasis on valuing the person, upholding personhood, meeting psychological and social needs, adopting the person’s perspective and ensuring a supportive social environment for people living with dementia is fundamental to social prescribing, thus potentially fulfilling a support need often not currently met [18] and increasingly utilised as part of holistic support packages for people with complex needs [19].

The effectiveness of social prescribing is difficult to quantify [20]. Benefits have been shown for people struggling with social isolation, mental health difficulties, and multiple health conditions [21–23], all issues impacting people living with dementia [24]. Some early evaluations showed positive effects on reducing GP and emergency department visits [25]. People living with dementia have benefited from a range of community activities, suggesting a social prescribing approach might help with the ‘non-medical’ aspects of living with dementia [26–28]. However, access to social prescribing services varies considerably in duration, intensity, and type of support offered, from telephone only contact and signposting to face-to-face assessments and involvement over weeks or months [15, 29, 30]. Also, how people access social prescribing impacts on the likelihood of service uptake [31]. The SPLW role provides a dedicated facilitator to support people, providing suitable referrals and motivating them to develop behaviour change [15]. Thus, SPLWs need in-depth knowledge of local community provision and to be trusted by the organisations they refer to [32]. Such skills and training were identified as critical for successful social prescribing but to date there is no particular qualification or experience required for the role [33].

In terms of dementia care, there has been limited research exploring the role of social prescribing, especially the variations existing between different service offerings and how these might impact people living with dementia. A recent study explored the consultation process between SPLWs and people living with dementia and found many challenges including communication weaknesses, particularly with telephone/virtual consultations, over dependency on the SPLW and a lack of knowledge about dementia in practitioners [34]. This raises questions about understanding what elements in a service offering might promote or hinder the ability of social prescribing to support people living with dementia. This study aimed to critically explore the current provision of social prescribing for people living with dementia across England, to identify which aspects of social prescribing services are most relevant for dementia care and support, with a focus on services tailoring their offering to a specific population.

## Methods

### Design

This study is part of a larger research programme developing and evaluating a dementia specific social prescribing intervention SPLENDID (National Institute for Health Research (NIHR) Programme Grants for Applied Research 203,280). The programme includes patient and public contributions that include a person living with dementia and carer who have input and oversight of research design.

A qualitative design enabled an in-depth understanding of the real-life experiences and perspectives of supporting people living with dementia within social prescribing services. Semi-structured, 1–1 interviews were undertaken with two groups across England: i) regional leads (managers) of social prescribing services to understand service structures and ii) SPLWs providing direct services to understand practitioner perspectives. Methods are reported in concordance with the Standards for Reporting Qualitative Research [35]. Ethical approval for the study was provided by the University of East Anglia (ID: ETH2324-1120).

### Recruitment

A purposive sampling strategy was used to ensure representation from regions across England and to ensure any variations across services were included. Potential participants were identified via three sources: i) the National Academy for Social Prescribing (NASP), ii) the National Social Prescribing Network (NSPN) and finally iii) snowballing by circulating recruitment advertisements via email. The aim was to interview up to 30 participants, with an equal balance of regional leads and SPLWs; including a mix of generic SPLWs and those working in specialisms potentially of relevance to people living with dementia, such as working with people living with dementia, older aged adults, or people with specific health conditions.

A regional lead was defined as anyone managing a social prescribing service based in England. They could manage other services in addition to social prescribing or be a lead SPLW who managed others; they did not have to be involved directly in service delivery. SPLWs were required to work in a service defined as ‘social prescribing’ in England and describe themselves as a ‘link worker’. Participants could be employed by NHS, voluntary, or community sector providers.

### Data collection

Interviews were conducted between September 2023 and February 2024 using either Microsoft Teams or Zoom by a researcher (AP) trained in interview methods using a semi-structured interview schedule developed by the wider research team (see Supplementary File 1). The schedule covered participant demographics, job role and region, social prescribing service delivery and structure in their area, thoughts on the social prescribing service offered to people living with dementia, and knowledge about dementia specific social prescribing in their area. Interviews lasted approximately 30 min and were audio and video recorded with additional field notes taken. Interviews were transcribed and anonymised, then the recording was deleted.

### Reflexivity statement

Interviews were conducted by a researcher (AP) with a background in community mental health practice. The wider team, experienced in qualitative data analysis, included a health services researcher, a clinical psychologist, two GP’s and a researcher interested in social care policy. The team has experience of working across primary and secondary care services and the charity sector. This collaborative approach ensured multiple viewpoints were considered, enhancing the credibility of findings. No researcher had prior relationships with participants.

### Data analysis

Data were analysed using template analysis to facilitate a comprehensive exploration of complex qualitative data [36]. Its structured approach is useful to maintain consistency across analysts, ensuring data are interpreted uniformly. Flexibility within this approach allows team members to collaboratively develop and refine the coding template, thus integrates diverse perspectives [36].

Analysis involved six steps [36]:

1. Familiarisation: A thorough reading through the data to become familiar with the content.
2. Preliminary Coding: Identify and mark initial themes or codes in the data.
3. Develop Initial Template: Create an initial coding template based on the preliminary codes.
4. Refine Template: Modify and refine the template as necessary.
5. Final Template: Finalise the template after thorough review and ensure all data are coded accurately.
6. Interpretation: Use the final template to interpret and analyse the data, drawing out key themes and insights

Steps 1–3 were completed by one researcher (AP). The coding template was then shared with two researchers (EW and LR) who refined the template. This was then shared with the wider team (JC, JR, KS, EW, AP) who each applied the template to a subset of transcripts. Data analysis clinics then occurred in person to review coding and interpret the data agreeing key themes and insights.

### Ensuring trustworthiness

To ensure the trustworthiness of the analysis, consideration was given to credibility, transferability, dependability, and confirmability throughout the research process. Credibility was supported through researcher triangulation, with multiple team members involved in developing and refining the coding template and interpreting the data collaboratively during in-person analysis clinics. Saturation was also considered in terms of the adequacy and richness of the data to fully explore the research questions, which was assessed through iterative analysis and ongoing team discussions during the coding process. Transferability was enhanced by using purposive sampling to capture a diverse range of perspectives from both SPLWs and regional leads across different regions in England, alongside rich contextual detail in reporting. Dependability was ensured through a clear and structured analytic process using template analysis, with an audit trail documenting coding decisions and template iterations. Confirmability was strengthened through team-based reflexive discussions and by maintaining awareness of potential biases throughout the analytic process, drawing on the varied professional backgrounds and experiences of the research team.

## Results

### Participant demographics

A total of 22 participants from six NHS England regions (London five; Midlands three; Northeast five; Northwest four; Southeast two; Southwest three) were interviewed. This included ten regional leads (service managers) and 12 SPLWs (service providers).

Participants had worked in the role between a few months to a few years. They had various backgrounds, some previously having worked in community roles, including homelessness, addictions, mental health, older aged adults and disabilities. Other previous experiences were in healthcare, education, customer service, and similar community navigation or link worker roles.

All regional leads (*n* = 10) managed social prescribing services but had different job titles. Some managed only social prescribing services and SPLWs, while others managed social prescribing alongside other areas. Many had a background working as a SPLW, and a small number still worked with patients as SPLWs in addition to their managerial role.

Of the 12 SPLWs interviewed, four were ‘generic’ working with people aged over 18 years. Eight had some kind of ‘specialism’ working exclusively or partly with a specific population. These ‘specialisms’ were: dementia specific where they worked with people living with dementia and their carers (2), older aged housebound adults (2), older aged adults (2), people struggling with mental health difficulties (1) and developing a dementia social prescribing project within their social prescribing service (1).

Four core themes were identified during analysis: family carer engagement key to supporting people living with dementia; service centred rather than person centred care; the dominance of dementia and strategies for success; dementia centred social prescribing.

## Theme 1: family career engagement key to supporting people living with dementia

Despite all participants indicating they, or their service, had worked with people living with dementia, many reported limited direct interactions with people living with dementia themselves, instead frequently describing their role as engaging with family carers. This provided direct support to family carers and harnessed the personal, detailed knowledge of their relatives, to enable better engagement with, and thus support, people living with dementia:

*“I think social prescribing is brilliant for dementia. I think you know an awful lot we can do for relatives. I think that's where the work is. It's supporting the families.” SPLW 10, Generic*

Almost all participants expressed a belief that supporting the carer ultimately supports people living with dementia indirectly, as the well-being of both were interconnected. By ensuring carers received the support they need, such as access to respite care, paid formal carers, or carer support groups, some discussed how social prescribing might enhance the quality of care provided to people living with dementia:

*“by supporting the carer, we’re supporting the patient as well.” SPLW 5, Older Adult*

Several factors contributed to this perception: a concern that people living with dementia may not fully understand, or be able to engage in, conversations and support; or that they may practically need carers to access referrals given by SPLWs, and a lack of confidence among participants regarding working with people living with dementia.

### People living with dementia may not understand the need for and/or engage in support

Many participants expressed that people living with dementia, particularly those in the later stages of the condition, may not comprehend the conversation around and/or the support being offered. Symptoms such as communication difficulties and/or memory impairment were cited as specific reasons for not working directly with people living with dementia; consequently, many practitioners resorted to using carers as intermediaries:

*“They're challenging remembering things, so quite often the work that that is done with people with dementia is by default more with their carer or family member” Regional Lead 3*

Some SPLWs wanted to speak with family carers to get a fuller picture of their relative’s support needs, concerned that if they spoke only to the person with dementia, they would refute the need for help, contradicting the carer’s narrative:

*“say if I speak to a dementia patient, they could say, “They're absolutely fine.” That doesn't mean they are. Like it doesn’t mean you get an accurate picture there. […] So, it’s like a lot of dementia patients say they're fine when they're not.” SPLW 5, Older Adult*

### Family carer: key facilitator to engage people living with dementia in support

Some participants shared it was necessary to work with the carer so people living with dementia could access the support required. Several participants highlighted some community activities might not accept people living with dementia unless accompanied by a carer; having a family carer was thus essential for referral. Also, the family carer may be needed to provide transport to the activities:

*“a lot of services won't have the person with dementia, unless they have a carer with them. So, they have that reassurance that they can manage them because they don't want to take that responsibility.” SPLW 1, Generic*

Some commented that family carer involvement was key to encourage and motivate people living with dementia to engage with community activities:

*“the main barrier is actually getting those referrals in the first place. Because I mentioned before, a lot of people with dementia will resist the support. So, actually you'll probably find that the way to overcome some of those barriers is actually through the carers.” Regional Lead 1*

### Lack of confidence interacting with people living with dementia

In many cases, SPLWs engaged with family carers regardless of whether carer involvement was essential for the referral process. Some indicated if they got a referral for a person with dementia they did not feel confident about, they might ask for the referral to be changed to the carer, or to add a separate referral for the carer allowing them to contact and communicate with the carer first:

*“some of the referrals I used to get, sometimes, you’d get a referral through for someone with dementia and it would look quite tricky. To be thinking, hmm, is this the best person to approach? And I would perhaps sometimes go back to the GP and ask how would you feel about putting the referral through for the carer? And then I would be able to speak to the carer” SPLW 12, Running Dementia Social Prescribing Project*

This uncertainty of how to work with people living with dementia was echoed by some participants who expressed feelings of nervousness when working with people living with dementia. In such cases, they commented that interacting with the carer provided them with a sense of reassurance. A regional lead reflected when they initially started working with people living with dementia this was often their reason for gravitating towards working with the carer:

*“I often used to work with the carers as opposed to kind of an individual with dementia because I think there was always that nervousness around what level of dementia somebody had.” Regional Lead 4*

## Theme 2: service centred, rather than person centred, approach

Large variability was found in support and services offered by social prescribing; how flexible a service offering was differed along with the kind of support SPLWs provided.

### Service constraints: how support is delivered and sustained

A key factor contributing to this service centred, rather than person-centred approach, was the options available to SPLWs in how they contacted and communicated with people living with dementia. Although most services afforded a range of communication options, including telephone, video calls and face-to-face meetings in a range of venues (GP surgeries, community settings, and home visits), telephone calls and face-to-face meetings in GP surgeries were most frequently discussed. Many SPLWs expressed that ideally meetings with people living with dementia should be done face to face in their homes to identify support needs that might be missed via telephone or away from the home:

*“it's kind of always preferable if you've got the kind of time to do that because it's like, “Oh, we're fine. We're getting on.” And then you go to the home and then you realise there's all these kind of issues and stuff.” SPLW 8, Dementia Specific*

However, the reality for many mostly generic SPLWs was time constraints often restricted their ability to provide home or face-to-face visits; some said this was a missed opportunity in providing good support for people with complex needs including people living with dementia:

*“We don't do huge numbers of home visits anymore just because of how things have changed since the pandemic and funding, and capacity constraints. So, I think that we wouldn't tend to see behaviours as much as if you were kind of seeing people more. And when we do see people face to face, it tends to be in community venues or at their GP surgery, where I think people who are able to access the community in that way often come with a bit of a different set of issues, a different mindset. Whatever it is you know it's not quite the same as that intimacy of being in someone's home when maybe they're a bit more relaxed.” Regional Lead 6*

In specialist social prescribing services there was greater variation in support, with participants discussing how the service offering was adapted to their target population for example, SPLWs working with housebound individuals understandably required more than phone calls to understand the needs of their patients:

*“we have to visit people in the home because some of the people that we interact with, they can't get out. They might be housebound, but they might not be very good on their feet. They can't walk very well. So, we go and visit them in the home. There are some people 65 plus who are mobile, obviously, I don't want to paint to stereotype here…. but yeah, most of the time we will visit people, well, all the time, really. On our first contact, we will visit people in the home.” SPLW 3, Older Housebound Adult*

One dementia specific SPLW highlighted that seeing people living with dementia in their homes had initially been considered best practice for this group. Unfortunately, in another service, increased caseload demand had led to cancellation of home visits and a return to the more usual approaches, i.e. telephone consultations, which hampered their ability to form closer working-relationships and support their patients’ needs:

*“I'm seeing them at home. We agreed very early on that in terms of best practice, the first visit would be done at home and then we would ascertain what works best for the patient” SPLW 7, Dementia Specific*

*“we were able to form really good working relationships with people, understand their needs, more, work more closely than a way which could help people to stay more independent for longer. So we would do more of that work. Unfortunately, as the caseload has gone up, we've had to fall back from that and go back to this more limited service.” SPLW 8, Dementia Specific*

The amount of time SPLWs could dedicate to people living with dementia varied, the duration of support, to a degree, was left to the discretion of the practitioner. Most participants emphasised that core to social prescribing was that it is a time-limited service (1–12 sessions) but this was often commented to be insufficient to support people living with dementia:

*“the amount of time I can spend with people is very limited on that. So my role is to initially phone people, once I get the referral through, have a bit of a conversation about what they need and trying either provide information, sign post people on to different services or refer people onto services as well.” SPLW 8, Dementia Specific*

*“I think that's the rule across the board with social prescribing really, is that it's about 12 weeks or 12 sessions. But I've gone over on mine loads of times and I’ve been able to say, you know, we're doing this, we're at this stage or we haven't achieved our goals. Because it's all about setting goals and it's about keeping the person as independent as possible, so you're not always going to achieve that in the time scale like you've got.” SPLW 4, Older Housebound Adult*

Some interviewees working in generic social prescribing services expressed that they were not always able to spend enough time supporting people living with dementia. In contrast those based in more flexible services were able to spend more time with patients, but still maintained a time-limited approach to social prescribing focusing on meeting patient goals. In contrast one dementia specialist SPLW provided indefinite long-term support until either the people living with dementia moved into a care home or died:

*“We always stay with them till they pass away or until they go into long-term residential care.” SPLW 7, Dementia Specific*

### Service-defined boundaries of support

Most generic SPLWs viewed their role as a facilitator, encouraging self-efficacy, fostering motivation, and signposting individuals toward appropriate services, rather than providing direct, ongoing support. The emphasis was on empowering people to take independent steps toward improving their wellbeing, rather than directly accompanying them to activities or offering hands-on assistance:

*“With social prescribing, we tend to prefer to signpost, just because it empowers the person, you know, it's more of a feeling of, OK, I've had this situation, but I've put it right. So you know it gives them more of a feeling of achievement if we can signpost as much as we can.” SPLW 9, Generic*

Services which had been tailored to support a specific population also stipulated an emphasis on empowerment and independence, however most roles went further than simply facilitating and/or signposting. They often had greater latitude to engage directly with people living with dementia in their homes or in the community, offering a level of individualised, ‘hands-on’ support that generic SPLWs could not, for example going for walks together or doing home-based activities. These were still considered core components of social prescribing, i.e. to empower and motivate, but through a more adapted and hands on approach:

*“I've got a lady at the moment who will just sit on her chair, and she gets up in the morning, sits on a chair. From the chair, she’ll go back to bed. So, we're dancing five-minutes each week, so that she's a bit more stable. She's got a little bit more confidence in her ability to walk, and I promised a coffee and cake on the sea front in May, if she can get her act together.” SPLW 7, Dementia Specific*

Despite specialist social prescribing services generally having greater flexibility, there was still considerable variation across and within such services, with clear constraints on the level of support they could provide and a definite emphasis on encouraging self-efficacy and self-motivation for people living with dementia to take up opportunities for themselves:

*“there are boundaries. You know, I wouldn't take people to the shops. I’m not allowed to get in my car. I'm not allowed to do any caring. Issues with them taking them to the toilet, things like that. No, I couldn't do things like that.” SPLW 3, Older Housebound Adult*

*“I often offer to take people to sessions. This wouldn't be the first introductory session. Once I’d gotten to know them a bit and I've got to know the hobbies and the likes and dislikes of the person living with dementia, I'll say, you know, if they want to join a walking group, I'll take them for the first session.” SPLW 7, Dementia Specific*

## Theme 3: the dominance of dementia in influencing support

Despite almost all participants stating that social prescribing could support anyone regardless of their medical condition, it was apparent that a diagnosis of dementia heavily influenced the support provided and importantly whether the SPLW considered people living with dementia suitable for social prescribing. Many struggled to think of ways to support people living with dementia, unsure what interventions might be suitable, and expressed that their needs would be better supported by a more specialist dementia service. This contrasted with the stated aim of social prescribing that the illness should not be the defining feature of the support given:

*“social prescribing is all about personalised care, because it is what matters to you as the individual, not what's the matter with you” Regional Lead 7*

Key aspects of dementia that negatively influenced the perceptions of ability to benefit included the nature of the dementia symptoms and the degree to which people had come to terms with, or even accepted, their diagnosis of dementia.

### Symptoms getting in the way

SPLWs expressed concern regarding whether they were able to offer beneficial support to people living with dementia in light of key symptoms such as memory impairment and the inability to make decisions. There was a perception that people living with dementia were more likely to have additional barriers to engaging in support including anxiety about visiting new places or trying new hobbies and embarrassment regarding attending dementia specific support:

*“if someone say is really struggling to keep track of their appointments or is struggling to kind of make decisions about next steps, then it makes it quite difficult for us to interact with that person in a really effective way.” Regional Lead 6*

*“when I have asked them, you know, I've checked with them, “Did you go to that?” and they go, “I never bothered.” So, I think sometimes you might put self-imposed barriers up themselves and not go to these things, you know. It could be out of they can’t be bothered or they feel embarrassed.” SPLW 3, Older Housebound Adult*

While these were potential barriers, some highlighted that there was a stage when a referral to any community activities could be unsuitable for people living with dementia for example, in advanced dementia, where a person could not remember the support offered and/or when their support needs were outside the SPLW’s capabilities. In such circumstances, the practitioner would end social prescribing support and refer the person with dementia back to their GP:

*“ …however fabulous those services are, there comes a point where people can't get there. Can't make an appointment to have a telephone call. Can't remember that they've been called. You know, there comes a point where however good the community services are, if you're elderly and living alone with dementia, there will come a point where that's just not enough.” Regional Lead 9*

*“then we call them and they go,” Hang on, who are you? What did we discuss?” And it's not quite that usual way that we have to remind and prompt people. We might then sort of take a bit more of a, not a hardline decision, but start thinking, OK, is this person actually benefiting from us being involved?” Regional Lead 6*

### Difficulties and dilemmas with referrals

Many had experienced people living with dementia being referred to social prescribing to help support them with their diagnosis. Consequently, two participants said that if a people living with dementia had not accepted their dementia diagnosis then they would not be able to support them, as they were to give dementia specific support:

*“we're trying to give them the support and encourage them to do these groups and go to the support, but they're very reluctant because they don't think that their diagnosis is that bad or isn't correct.” Regional Lead 5*

Also, many stated they found it difficult to decide which interventions would be suitable for people living with dementia, this was the case for both generalist and some SPLWs working with older adults also:

*“I think with like people I work with who don't have dementia, it comes very like oh, I’ll refer to there, I’ll refer to there, whereas I think with dementia, a lot more thought is going into it.” SPLW 5, Older Adult*

When referring to non-dementia services, some participants said this may not be accessible for people living with dementia, particularly if their symptoms were more advanced and some services would not accept anyone with dementia regardless of the stage of the illness:

*“Yeah, they just sort of say, “Oh, well, volunteers don't have the skills to manage.” And it just seems a bit of a shame and a bit of a blanket reason, when actually, dementia presents in quite a lot of ways” Regional Lead 3*

Some participants commented that the needs of people living with dementia were more ‘clinical’, and outside their ‘social’ remit; they discussed how there was little support they could give other than passing on to dementia specific services. They also referred to dementia specific support if the SPLW was struggling to work with a people living with dementia, or feared they were doing more harm than good, feeling that someone with more knowledge of dementia might be better placed to offer help:

*“we aren't clinicians, and we don't have that insight into kind of something specific, it's about kind of how best we work with an individual to kind of connect them in with appropriate services. We aren't there to fix it. We are very much there as a sort of signposting on to the next kind of group or organisation.” Regional Lead 4*

*“She couldn't communicate with me. Her husband was finding it really difficult and I just felt it was better that I wasn't there and I referred it to the Alzheimer’s Society because I found that I was probably stressing her out as well, by going. Because I was this stranger just turning up.” SPLW 4, Older Housebound Adult*

However, one SPLW discussed the conflict they experienced not being able to support a person with dementia once they had been referred to a dementia specific service, feeling they were not able to offer as much support as they would like:

*“for example, your job is to refer them to Dementia UK and make Dementia UK’s job to support them, you know. That's the way my company see it. I try to see it different.” SPLW 10, Generic*

## Theme 4: strategies for success: dementia-centred social prescribing

Many participants described considerable conflict between trying to deliver a theoretical, person-centred, model of social prescribing to a patient group living with an illness most SPLWs had little knowledge and experience of, within existing social prescribing structures and frameworks that were not a ‘natural fit’ for dementia care. Notwithstanding this, some did develop and demonstrate strategies for successful ways of engaging with people living with dementia through their personal and/or professional knowledge and experience of dementia.

### Benefit of personal and/or professional experience of dementia; seeing beyond dementia

Importantly, participants shared that the perception that dementia always required specialist support was a common but unfair assumption; however, they agreed it could mean people living with dementia were not always supported as much as they could be. This was more common in participants with more experience of dealing with dementia, either in their own family or via professional experiences. These participants were aware of the possibility of making assumptions about mental capacity or the activities people living with dementia could enjoy or engage in. One SPLW who worked with older adults suggested people might make such assumptions before meeting people living with dementia because of seeing the word dementia:

*“I think that people with dementia are massively underserved because there is that feeling of needing something to be quite specialist.” Regional Lead 6*

*“I've had someone whose had their diagnosis since 2014, and thinking, gosh, it must be quite far along and their memory must have really deteriorated. And then I did the review with them, and …I’m like, ‘Oh my gosh, I wouldn't even think they had dementia, if I didn't see that or know that.’ And then he went to some like groups … and engaged really well. So, sometimes I think you just see the word dementia and you can think, oh, they can’t remember anything. [Laughs] I’ve learnt that you can’t go on what you think.” SPLW 5, Older Adult*

Some suggested they tried to not make assumptions about the capabilities of someone because they saw the word dementia, but rather used personal skill and judgement to decide whether someone might benefit from social prescribing support:

*“there’s also a lack of understanding because, you know, if someone’s sort of…if the behaviour is being aggressive, you don't know how to deal with it because they might not know about dementia. Whereas if you've got a better understanding, you know, sort of work with the person better.” SPLW 4, Older Housebound Adult*

In addition, some largely specialist SPLWs mentioned tailoring their communication techniques and consultation approaches to adapt to difficulties related to the dementia, such as writing things down, doing text follow ups, and getting people to repeat information back to them to ensure understanding, thus dementia symptoms did not preclude them benefiting:

*“So if you write something down, especially with dementia, and then you have this piece of paper on the table on a fridge, this then might trigger your memory and will remember what this was all about. So I always take time with people to make sure, they write things down, ask them to repeat back and summarise at the end.” SPLW 9, Generic*

Other techniques included focusing on personal interests to maintain engagement or utilising eye contact or touch to create a bond:

*“I was chatting to her husband. And the lady was finding it really difficult to communicate with me and her husband says, “Oh, you know, she's having some problems with her memory.” So, I said “OK.” so I try to focus on the things that she likes. So, I said, “So, what music did she used to like?” And she said she liked the Eagles. And I said, “Oh, I said, I love the Eagles. I went to see them in concert.” And we just straight away, we had like this little connection and it was lovely.” SPLW 4, Older Housebound Adult*

*“quite often you sit next to the person who's got dementia, because that way I've got a better eye contact. If their hearing's poor, I'm right beside them and a hand will come across and you've made a connection already because that person feels able to trust you.” SPLW 7, Dementia*

Those who worked largely with people living with dementia said they found it helpful to undertake separate consultations with people living with dementia and their carers. They reported making a concerted effort to speak with the person with dementia alone to build rapport and encourage them to express their own preferences. This brought balanced input from both carers and people living with dementia, ensuring the support provided aligned with needs and capabilities:

*“we're really passionate about ensuring that the person with dementia has a voice and we listen to what they feel like.” SPLW 1, Generic*

*“one of the things you learn very early on is a bit of separation, so I often send the carer off, “Oh, I'd love a cup of tea.” So, you can have a chat with the person with dementia because they become quiet when there is somebody they deem to be professional, and they let their partner do all the talking. But once you get them chatting. I mean I hoping to set up a gardening group with a local first school and one of the guys I saw this week, he's a real keen gardener, but he didn't tell me that while his wife was there because his wife was doing all the talking. But once we got rid of her, he was sort of telling me what he liked to do.” SPLW 7, Dementia Specific*

### Opportunistic approaches: the annual dementia reviews

Participants discussed how they identified opportunities to engage people living with dementia through being involved in their usual medical care. In England, GPs are required to undertake an annual medical review on all their patients with dementia. One SPLW who was conducting a review into how their social prescribing service could better support people living with dementia, discussed how involvement of SPLWs in dementia reviews could bring benefits. They discussed how GPs were unsure how to support the non-medical needs and that involving SPLWs could be a more holistic approach:

*“I realised that they were entitled to these annual dementia reviews, I kind of started thinking, well, why on earth are things not being picked up on these reviews? And when we scratched beneath the surface, we realised that the people that were conducting the Annual Dementia Reviews, felt like they weren't doing a good job. A very honest, GP said, “We don't really know what to do. We don't know what to do with people with dementia. It’s kind of not my thing and we don't know what's on offer out in the community.” SPLW 12, Running Dementia Social Prescribing Project*

Other SPLWs discussed their involvement in annual dementia reviews either contributing to the review process or advertising their role during them:

*“at the end I pop in and say, “Hello. This is me and this is how I can support you”. SPLW 7, Dementia Specific*

SPLWs who participated in, or conducted, annual dementia reviews used the opportunity to explore non-medical needs that might otherwise remain unexplored in a medically focused consultation. One dementia specific SPLW found her attendance at these long-term condition reviews resulted in every patient at one GP surgery taking up social prescribing support when the introduction was made:

*“I'm going along to the long-term condition review for people that have got a dementia diagnosis and if I know them, it's five-minutes, “Hello, how are you?” If I don't know them, they're offered a home visit while they're in the surgery, and so with one surgery, we've had 100% take up of that offer through long-term condition review.” SPLW 7, Dementia Specific*

During dementia reviews participants emphasised they communicated both with the people living with dementia and the carer, illustrating both commonalities and variations in how different social prescribing services are trying to support people living with dementia:

*“usually when doing dementia reviews and working with people that have dementia. I communicate with them and the family, in making sure they have got everything they need.” SPLW 11, Older Adult*

## Discussion

### Summary

We set out to explore how social prescribing services across England provide support to people living with dementia by exploring the offering provided by SPLWs working with general or specific populations. This showed how SPLWs gravitate towards working with family carers, rather than directly with people living with dementia often due to a lack of confidence and expertise in dementia care. However, this assisted SPLWs as the family carer provided ‘expert patient knowledge’ and acted as a facilitator between them and the people living with dementia. The structure and framework of social prescribing services plays a role in how support is delivered, with greater flexibility in the SPLW’s role and responsibilities enabling more person-centred dementia care. Although participants strongly advocated social prescribing should be available to anyone regardless of medical issues, many found supporting people living with dementia challenging, especially participants with limited experience working with people living with dementia. SPLWs who had the capacity, knowledge, and flexibility in their role to adapt their approaches reported greater success both in the quantity and quality of support provided. In addition, opportunistic integration of SPLWs into existing primary care services, such as annual dementia reviews, provided closer involvement and opportunity. These findings highlight the tension between the person-centred ideals of dementia care and social prescribing and the reality of a limited workforce supporting people living with dementia within a resource-constrained care system.

### Comparison with existing literature

A key finding was SPLWs often engaging with carers rather than directly with people living with dementia. Although this suggests not involving people living with dementia in their care, there were also benefits, such as directly supporting the family carer and also facilitating engagement and closer working between the SPLW and the people living with dementia. This is consistent with the wider literature on the responsibilities of caring for people living with dementia and the important role of families in dementia care [1, 11, 37–39]. Notwithstanding, while carer support may indirectly benefit people living with dementia, our findings raise questions about the primary beneficiaries of social prescribing and whether current service models are adequate to directly meet their needs [34].

Recent research on social prescribing consultations with people living with dementia identified a lack of knowledge and experience of dementia as a critical factor in how SPLWs engage with this group, especially those with more advanced dementia [34]. This aligns with previous research involving health and social care practitioners who often feel underprepared and under-skilled to work with this population, negatively influencing practitioner-patient interaction [40]. In addition, this could also restrict opportunities for people living with dementia to participate in decisions about their care and interventions which could improve their wellbeing [41]. The non-clinical nature of most SPLW roles raises questions about the extent to which they are equipped to support people with complex conditions such as dementia without additional training, or working alongside others with more specialist expertise, especially as our findings show the latter to have skills and strategies to better involve people living with dementia. These approaches reflect ongoing calls for more integrated, person-centred practices in dementia care [11].

A significant issue was the tendency for SPLWs to adopt a “diagnosis-first” perspective, where support was focused on what could be done to support dementia symptoms or difficulties, rather than a person-first perspective. This phenomenon is common in dementia and limits person-centred approaches, reinforcing stigma [3]. In social prescribing, this risks people living with dementia missing the potential wider benefits of a model that aspires to offer holistic and personalised care. However, those with more knowledge of dementia, either through personal or professional experience, seemed better able to see ‘beyond’ the diagnosis. Older adult or dementia specific SPLWs demonstrated greater confidence in communicating directly with people living with dementia, were more confident at assessing mental capacity and tailoring their approach accordingly. This reflects literature which emphasise the importance of practitioner familiarity with the nuances of cognitive impairment in facilitating more inclusive and person-centred support [40]. Specialist SPLWs also appeared more able to recognise and respond to personal interests and abilities, providing person-centred dementia care [3].

### Implications for practice and future research

SPLWs consistently described their role as focused on motivation and information provision, and as gatekeepers to other services, consistent with international models of social prescribing that emphasise autonomy, goal-setting, and short-term interventions [15]. However, social prescribing services face distinct challenges in supporting people living with dementia, both due to the direct impacts of the condition and indirectly because of limited availability or accessibility of appropriate community support. Despite recognising these barriers, many services lacked the flexibility or resources to address them effectively. This suggests a need for clearer guidance on ways social prescribing is expected to support people that enables more tailored, sustained approaches to support people with complex and fluctuating needs. Services that offered more flexibility, often tailoring their support to a specific population, seemed to offer more holistic provision, with practitioners with a better understanding of dementia finding ways to make social prescribing work for people living with dementia, suggesting that dementia-specific workforce development is essential. In line with previous findings [34], SPLWs may benefit from targeted training in dementia-friendly communication, assessing and supporting decision-making capacity, and understanding the progression and psychosocial impacts of dementia.

The findings suggest that a universal approach may disadvantage individuals with more complex needs, raising concerns about equity within current social prescribing models. A model that relies heavily on individual agency may be poorly suited to people with cognitive or functional limitations that affect their ability to engage without sustained support. This systems-led approach reflects broader critiques of fragmented service design in health and social care [42] and highlights how structural priorities such as autonomy conflicts with the complex, ongoing needs of individuals with long-term conditions. Thus, different approaches/models such as proactive SPLW engagement in annual dementia reviews, longer-term or relational forms of working, and closer integration with primary care or memory services, warrant further exploration. These models may be better aligned with the needs of people living with dementia and should be evaluated for feasibility and impact in future research.

### Strengths and limitations

Key strengths are the inclusion of both service managers and service providers. Participants from a variety of social prescribing services, including dementia-specific and non-dementia-specific services, and from different settings such as general practice and the third sector, facilitated a range of perspectives. The online interviews facilitated in-depth flexible exploration, capturing personal experiences, challenges, and practical concerns including insights into real-world constraints in social prescribing roles. This provides a rich understanding of how social prescribing is functioning for people living with dementia across different contexts. The inclusion of SPLWs with personal connections to dementia through previous work or family members, adds depth. This variation in experience and service structure was valuable but makes it difficult to draw wider, more generalisable conclusions regarding how social prescribing is supporting people living with dementia at a national level. Significant limitations are that participants were self-selecting, suggesting those with a particular interest or experience in the topic may have participated. Additionally, and importantly, the scenario of people living with dementia living alone was notably absent from most accounts. This is a concern as prior research indicates people living with dementia who live alone are less likely to receive community-based support, often due to the absence of an informal carer to facilitate access [4]. Also missing was any discussion of potential cultural or language barriers to accessing social prescribing. This could reflect lower engagement with social prescribing among these groups or may indicate hidden barriers to accessing support that SPLWs may be unaware of, highlighting the need to further understand how issues of culture, language, and equity are understood within social prescribing services.

## Conclusions

While the involvement of carers may at times be needed for practical reasons, SPLWs’ limited interaction with people living with dementia reflects broader challenges in addressing the specific needs of people living with dementia within the current social prescribing framework. SPLWs’ success in supporting people living with dementia with social prescribing depends on how well the needs of people living with dementia matched the structure and constraints of existing services and whether any flexibility was built into the system. Successful strategies to improve engagement with people living with dementia included adapting contact and communication methods. The combination of expert knowledge and more role flexibility to enable home visits, longer support times and involvement in medical reviews, enabled largely specialist SPLWs to provide a different support offering for people living with dementia. As ageing populations lead to increasing numbers of people living with dementia, social prescribing has the potential to be an important component of post diagnostic dementia care within resource strapped healthcare systems where specialist services are declining or very limited [11, 39]. Whilst this study suggests a specialist SPLW model may be more appropriate and effective for people living with dementia and generalist SPLWs require additional dementia training, further research is required to explore whether such variations within social prescribing make a significant difference to the health and wellbeing of people living with dementia and in particular those who do not have a family carer to support them.

## Supplementary Information

Supplementary material 1.

## Data Availability

The transcripts analysed during the current study are available from the corresponding author on reasonable request.

## Abbreviation

SPLW: Social prescribing link worker
