# Supplementary material for: Social prescribing in primary care for people living with dementia: a qualitative exploration of different roles and services in England
Source: BMC Prim Care. 2025 Nov 7;26:346. doi: 10.1186/s12875-025-03011-9 (PMC12595909; doi:10.1186/s12875-025-03011-9)
Supplement: Supplementary file 1 — Supplementary material 1. [file 12875_2025_3011_MOESM1_ESM.docx]

# Interview Guide

## Introductions

1. Introduction and explain aim of the interview is to understand current social prescribing provision and support for people living with dementia
2. Explain that we simply want their opinions and experiences from their area, and that there are no wrong or right answers
3. Check consent for recording is given and check consent form has already been gained via email, if not then read consent questions individually and gain consent verbally
4. Explain length of interview will be limited to 30 minutes, unless they wish to talk for longer

## Questions

**Demographic questions:**

We will start by asking you to tell us a bit about yourself and your role.

- What is your job title?
- Roughly how long have you been in this role?
- What type of roles have you worked in in the past?
- What region/area do you cover?

Now we’ll ask a bit more about social prescribing and people living with dementia.

1. **What structure does Social Prescribing take in your area?**

Prompts:

Does social prescribing have to have Link Worker involvement?

Could a Link Worker role also have a wider function, i.e. a care navigator who supports with care planning and additionally gives social prescriptions?

Where do you see most referrals coming from? Primary care workers or other organisations? Is this the same for referrals of People Living with Dementia?

1. **Can you tell us your thoughts on what, if any, value social prescribing might give to people living with dementia and their carers?**

Prompts:

In your opinion could it offer benefits, and if so what?

Can you think of any reasons why social prescribing might not be of benefit to people living with dementia and their carers?

Do you think people living with dementia might gain something different from Social Prescribing (to people without dementia)?

1. **In your experience what, if any, barriers to social prescribing might people living with dementia face?**

Prompts:

Are people living with dementia just as able to access social prescribing as others?

As far as you know, what involvement do carers have in Link Worker discussions for people living with dementia?

Do you know if many referrals for people living with dementia come through?

Do you feel Link Workers are able to work productively/successfully with people living with dementia? If yes, then why? If not, then what might help them (e.g. dementia specific training, knowing of dementia tailored activities, etc.)?

What changes (if any) do you think would have to be made in the services on offer for it to be more suitable for people living with dementia?

1. **What, if any, Social Prescribing services in your area are offered to people living with dementia?**

Prompts:

As far as you’re aware are most services open to people living with dementia (even if not specifically designed for them)?

Do you know of any services that offer tailored Social Prescribing dementia services?

If so, then do you know what is tailored about the service (e.g. involves carers and/or family, trained Link Workers, links to dementia specific groups etc.)?

**Would you be able to give me contact details for these services so I can contact them about the next step of our research? (prompt question to collect potential participant contact details for Step II).**

## Conclusions

1. Conclude interview
2. Signpost to SPLENDID website and any websites giving information about dementia (details on Participant Information Sheet)
3. Ensure contact details for any dementia specific services have been obtained
